# The Multi-Scale Modelling of Coronary Blood Flow

**DOI:** 10.1007/s10439-012-0583-7

**Published:** 2012-05-08

**Authors:** Jack Lee, Nicolas P. Smith

**Affiliations:** Department of Biomedical Engineering, King’s College London, King’s Health Partners, St. Thomas’ Hospital, London, SE1 7EH UK

**Keywords:** Coronary blood flow, Multi-scale modelling, Multi-physics

## Abstract

Coronary flow is governed by a number of determinants including network anatomy, systemic afterload and the mechanical interaction with the myocardium throughout the cardiac cycle. The range of spatial scales and multi-physics nature of coronary perfusion highlights a need for a multiscale framework that captures the relevant details at each level of the network. The goal of this review is to provide a compact and accessible introduction to the methodology and current state of the art application of the modelling frameworks that have been used to study the coronary circulation. We begin with a brief description of the seminal experimental observations that have motivated the development of mechanistic frameworks for understanding how myocardial mechanics influences coronary flow. These concepts are then linked to an overview of the lumped parameter models employed to test these hypotheses. We then outline the full and reduced-order (3D and 1D) continuum mechanics models based on the Navier–Stokes equations and highlight, with examples, their application regimes. At the smaller spatial scales the case for the importance of addressing the microcirculation is presented, with an emphasis on the poroelastic approach that is well-suited to bridge an existing gap in the development of an integrated whole heart model. Finally, the recent accomplishments of the wave intensity analysis and related approaches are presented and the clinical outlook for coronary flow modelling discussed.

## Introduction

**Figure 1:**
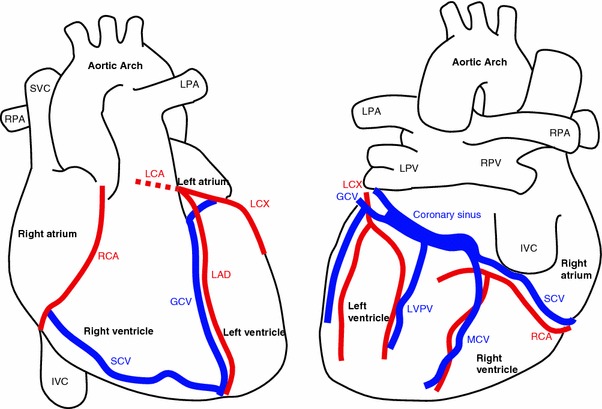
Anatomy of the heart and major coronary vessels in anterior (left) and posterior (right) orientations. Coronary arteries: left coronary artery (LCA), left circumflex (LCX), left anterior descending (LAD), right coronary artery (RCA). Coronary veins: coronary sinus (CS), great cardiac vein (GCV), middle cardiac vein (MCV), small cardiac vein (SCV), left ventricular posterior vein (LVPV). Pulmonary vessels: left and right pulmonary arteries (LPA/RPA), left and right pulmonary veins (LPV/RPV). Vena cava: superior vena cava (SVC), inferior vena cava (IVC)

**Figure 2:**
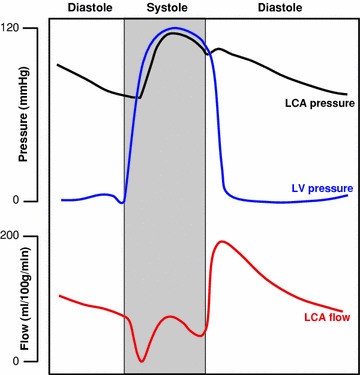
Wiggers diagram of a typical human heart. Shown are pressure and flow in the left coronary artery, together with the left ventricular pressure. LCA flow is impeded during systolic phases due to contraction of the myocytes and the transmitted pressure from LV. The pressure in the LCA is maintained into diastole, where most arterial flow occurs, and depends primarily on the systemic afterload rather than LVP since the aortic valve is closed

Over the past decades, technological and clinical driving forces have fuelled significant advancements in modelling of blood flow in the coronary vessels of the heart. However, while the governing models and numerical solution techniques are now well-established, the complexities introduced by the anatomy and the dynamic mechanical environment produced by heart contraction present a number of further technical challenges. As emergent models of large-scale coronary circulation have demonstrated, a multiscale modelling approach in which heterogeneous modelling strategies are employed at different network scales has the potential to be both physiologically accurate and computationally effective. This review aims to provide a compact summary of selected past and recent state of the art in coronary modelling, extending over full and reduced-dimensional approaches relevant for constructing a multiscale model and where appropriate, link these developments to clinical applications. For the benefit of the general audience, readers may refer to the basic coronary anatomy (Fig. 1) and hemodynamic characteristics (Table 1, Fig. 2) as background to this review.

**Table 1:** Overview of coronary vessel characteristics

Vessel type	Diameter	Wall composition	Diameter variation (diastolic-systolic)	Peak velocity	Notes
Major arteries (LAD, RCA etc.)	3–5 mm	Full 3 layers (Lumen:Wall ~ 3.5)	~15%	~40 cm/s	<10% total coronary resistance
Arteries	>300 *μ*m	Full 3 layers	–	–	Intramural arteries supply the subendocardium
Small arteries	150–300 *μ*m	4–6 smooth muscle layers	–	–	Major site of resistance
Arterioles (muscular)	20–150 *μ*m	1–4 smooth muscle layers	25/3% (subendo/subepi) (dog)	~4 cm/s	Arterio-luminal communications^a^
Terminal (precapillary) arterioles	10–20 *μ*m	Pericyte layers replace smooth muscles	–	~7 mm/s (dog)	Inter-distance of 700 *μ*m: encloses a microcirculatory unit, which also contains a postcapillary venule
Capillaries	5–10 *μ*m	Endothelium and basement membrane only, fenestrations	Does not completely collapse	1–4 mm/s (dog)	Exchange function Capillary sinuses^b^
Venules	10–50 *μ*m	Pericyte layers replace smooth muscles	~10%: endo-to-epi shift during systole (dog)	~7 mm/s (dog)	Major site of exchange
Small veins	50–300 *μ*m	1–3 smooth muscle layers	–	–	Reservoir function
Veins (muscular)	>300 *μ*m	Full 3 layers	–	–	Thebesian veins^a^
Greater veins (GCV, MCV etc.)	1–7 mm	Full 3 layers	–	~13 cm/s (~40 cm/s dog)	Drains into coronary sinus
Coronary sinus	~10 mm	Full 3 layers	12–42%	~2 cm/s (mean)	Drains into right atrium

The classification of vessels by diameters indicated here are broadly based on anatomical and functional criteria and are representative of those found in the literature. The shown figures were, as much as possible, obtained from studies in the human coronary circulation. As the transition from an artery to an arteriole is gradual without abrupt transitions, the threshold diameter of 300 *μ*m selected for microcirculation is arbitrarily selected within the usual range of 100–400 *μ*m. The three layers of the general vascular wall consists of intima (endothelium), media (smooth muscles) and adventitia (fibrous connective tissue), and vessel types vary most notably by the content of the medial layer. Due to the high intra- and inter-subject variabilities, the velocities and diameter variations listed are indicative of the order of magnitude only, under normal conditions

^a^Special hemodynamically relevant structures: direct communication from arterial and venous vessels with the cardiac chambers

^b^Capillary and precapillary sinuses: reservoir-like spaces within the capillary network that could act as micro-pumps

The majority of early coronary models employed lumped parameter frameworks to postulate and test the key mechanisms by which flow is determined. Often closely integrated with experimental work, these studies set the stage for initial understanding of the origins behind the pulsatile and phasic nature of the coronary flow and hemodynamic forces governing it. In particular, these pioneering research efforts reinforced the importance of the coronary-myocardial coupling (termed by some as ‘crosstalk’) and that, in order to understand the coronary circulation, an integrated quantitative approach is indispensible.

**Figure 3:**
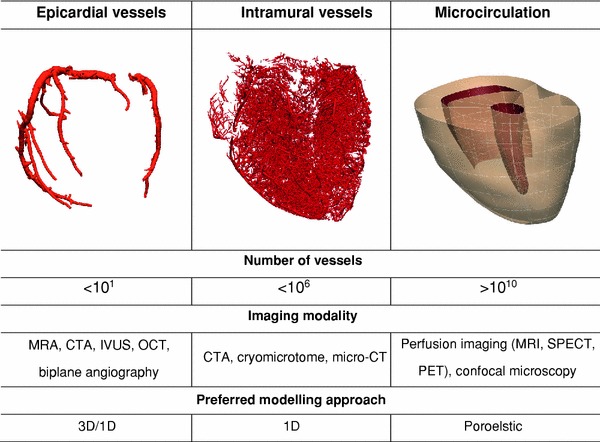
A schematic of the different anatomical scales with associated imaging modalities and modelling approaches applied to construct and simulate coronary blood flow respectively

The more recent models of the coronary circulation have increasingly leveraged the parallel developments in structural and functional imaging technology that provided increasingly detailed computational domains and boundary conditions, as well as the improved solution algorithms and computing power. These technologies have enabled the construction and solution of larger integrated models through the application of three broadly different approaches reviewed herein (see Fig. 3). The first of these is one-dimensional network modelling, often employed to study wave propagation phenomena in a spatially distributed coronary bed, which is, in turn, significantly affected by the transmural variation in myocardial interaction. The second approach builds upon the ongoing computational advances that have rendered detailed three-dimensional computational fluid dynamics (CFD) increasingly tractable, and is relevant to image-based diagnosis and interventional planning as well as exploring factors underlying atherosclerosis development. Finally, the poroelastic modelling approach has received less attention than the previous modelling frameworks, but is well suited for studying the bilateral interaction between coronary flow and myocardial deformation, thus readily lending itself for whole-heart cardiac function modelling.

Although clinical applications of the above-mentioned methodologies have only recently become a major focus for the modelling community, those based on wave intensity analysis have already yielded successful outcomes. As described below, the current established measure for functional diagnosis of stenosis severity (fractional flow reserve) has seen limited uptake in clinics in spite of the overwhelming clinical supporting evidences, owing to its practical difficulties. A new index (instantaneous wave-free ratio) based on the theory of wave intensity analysis introduced recently appears well-posed to address some of these drawbacks, and serves as a noteworthy example of bench-to-bedside translation.

This review is organised as follows. First, the key historical developments in lumped-parameter modelling of coronary flow are reviewed, with an emphasis on the experimental findings which motivated the development of specific frameworks. Progressing from this historical context, three different continuum mechanics approaches are described, including the one-dimensional and three-dimensional computational fluid dynamics, and poroelastic modelling of blood flow in tissues. Finally, the role wave intensity analysis has played in clinical developments is visited as a contemporary example of the potential of modelling technology, and a motivation for its developmental outlook.

## Earlier Concepts in Coronary Modelling

The coronary circulation is distinguished from other vascular networks by its phasic flow pattern.^81^ Most of the coronary arterial flow occurs in diastole, while during systole the arterial flow may slow down or even reverse, while the venous flow accelerates. These out-of-phase transient changes are not caused by fluctuations in the coronary perfusion pressure but, rather, are broadly attributed to the interaction of coronary flow with cardiac contraction termed ‘mechanical crosstalk’. The exact mechanisms of crosstalk had been under much investigation (For comprehensive reviews of the mechanisms, refer to Spaan^89^ and Westerhof *et al.* 109). Earlier attempts to understand the mechanical determinants of coronary flow deployed single predominant mechanism in a lumped-parameter model to explain the then-available experimental data. A selection of ideas which steered the major developments in the field is reviewed below.

### Waterfall and Intramyocardial Pump Models

The concept that distal vessels collapse due to the compression of the surrounding myocardium led to the vascular waterfall model.^28^ Based on the previous work on flow in collapsible tubes,^40^ it was assumed that when a vessel partially collapses, the flow is no longer a function of the pressure gradient between the arterial and venous pressures; but rather it is the gradient between arterial pressure and the intravascular pressure at the collapse point that determines the flow. With a 4-transmural layer lumped elements, this simple model was able to reproduce the measured arterial inflow. However, the shortcomings of the waterfall model are apparent when the venous flow is considered. In its proposed form, when the vessel collapses the flow eventually halts completely without exhibiting the experimentally-observed inflow reversal or outflow augmentation. To address this issue, the intramyocardial pump model^90^ was proposed to characterize the mechanism of systolic vascular compression leading to displacement of fluid. This model highlighted the importance of the vascular capacitance which serves to store the fluid during diastole which then is actively pumped away by the myocardial contraction. Many subsequent models were proposed based on the waterfall and intramyocardial pump mechanisms.^2^,^11^,^12^,^18^,^113^,^114^

### Varying Elastance Model

Most of the models mentioned above share the common assumption that the pressure to which the vessels are subjected within the myocardium (intramyocardial pressure or IMP), decreases linearly from the left ventricular pressure (LVP) at the endocardium to zero pressure at epicardium. This association of LVP to vascular compression was debated on grounds that measured IMP may sometimes exceed LVP,^56^ IMP is confounded by the compartmentalization of pressures in various microstructures within the tissue and also depends on the local venular emptying rate.^89^,^110^ Subsequently, the pivotal evidence which supplanted the assumptions underpinning these previous models was provided by the experiments which compared coronary flow in a contracting myocardium with pressurised (isovolumic) or non-pressurised (isobaric) ventricular chambers. That the flow in either case was similar, in both isolated^54^,^55^ and *in situ* 102 setups, indicated that another mechanism must be responsible for the systolic flow impediment. This led to the introduction of vascular varying elastance model,^54^ based on the concepts originally applied to the cardiac chambers.^92^ In this model, the increased stiffness of the myocardium during systole was postulated to drive the coronary flow through modulating the compliance and resistive properties of the embedded vessels. At low levels of LVP, the contracting ventricle was observed to prevent the LVP from being transmitted in the myocardial wall, thereby shielding the intramural coronary vessels from compression.^53^,^71^ Further experiments confirmed the dissociation between LVP and systolic impediment by conversely showing that, with an impaired regional cardiac contraction (*via* application of lidocaine) coronary flow exhibits a considerable dependence on the peak LVP.^27^

### Lumped Parameter Models and Integrated Modelling

While each of the above experimental studies has contributed elegant mechanistic hypotheses to explain the origin of coronary flow impediment, collectively they show that no single measurable index or mechanism is solely responsible for the phasic flow patterns. This determinant is now understood to be the summed contributions from coronary pressure, the direct (myocardium-vascular interaction) and the indirect (through IMP generation) crosstalk, modulated by contractility, transmural position, afterload, heart rate and the diastolic time interval. Accordingly the lumped parameter models proposed following these seminal studies have been integrative, for example, coupling LV mechanics, coronary flow and fluid and mass transport to test the mechanisms of coronary compression,^113^ or the shielding effects of vasculature at low LVP.^9^ Also, the integrative approach has been used to investigate aortic stenosis.^34^

With a few exceptions, the majority of recent coronary modelling studies have employed a continuum mechanics approach, which is discussed in subsequent sections. Nevertheless, the lumped-parameter approach remains a useful element in the multiscale vascular modelling for retaining the computational tractability, as demonstrated by a number of recent works.^30^,^49^,^51^,^67^,^87^,^88^,^101^

## One Dimensional Flow Modelling

The key feature of the one dimensional modelling over the lumped parameter approach is the ability to incorporate wave propagation phenomenon. Through a comparative 1D vs. lumped parameter model study, the importance of the wave phenomenon has been demonstrated, particularly pertaining to systolic compression.^38^ The 1D model also offers significant computational savings compared to simulating full 3D CFD, while capturing the pulse wave characteristics of long wavelength and cross-sectionally averaged flow and pressure in deformable vessels. It is therefore the currently preferred approach for vascular modelling in networks of many vessels. In the following sections, key aspects of the 1D approach are reviewed.

### The Standard Model

The standard 1D flow equations used in many models^14^,^32^,^60^,^68^,^70^,^77^,^83^,^84^,^88^,^104^ to describe the conservation of mass and momentum can be obtained by averaging the Navier–Stokes equations over the cross-section of an axisymmetric circular cylinder,^88^ or derived from first principles.^83^ A characteristic analysis of the governing PDE system shows that it is hyperbolic, with forward and backward traveling waves.^32^ As these equations model the flow in a deformable vessel, the interaction between fluid and vessel wall warrants further description, requiring an additional equation. The system closure is usually achieved by assuming an algebraic form of the pressure-area (p-A) relationship, derived from a linearly elastic shell model^32^,^83^—which will “blow out” at high pressures, but is deemed valid for physiological operating conditions—or otherwise empirically formulated.^88^ Several forms of the p-A relations were also proposed for the collapsed regime, exhibiting large compliance at first but becoming rapidly stiff toward total collapse point.^38^,^88^ The viscous momentum loss in this system is often represented as a force term, which is often derived from an assumed shape of velocity profile across the cross section.^88^ Alternatively, a flat profile with a linear boundary layer has also been used.^70^

### Boundary Conditions

The inflow conditions in many 1D modelling studies use waveforms directly measured from experiments^38^,^88^ or generated by a simplified model of the heart,^68^ presuming a dominant role of aortic pressure over coronary perfusion. In contrast, much attention has been paid to formulating the outflow condition, which must capture the characteristics of the distal microcirculation network. The simplest outflow condition is to directly prescribe a pressure or pressure-dependent flow at the distal terminal. However, this is undesirable since it introduces spurious reflections back into the computational domain, fails to reproduce the phase lag between the reflected pressure and flow, and ignores the considerable compliance of the microcirculation. A windkessel outflow condition (reviewed in Westerhof^111^) offers improvements by incorporating compliance as well as resistance and thus exhibits the frequency-dependent reflection coefficients, although not necessarily in a physiological fashion particularly at higher frequencies. To address these issues, an alternative approach was proposed where the input impedance of the distal network was derived using a generalized branching network.^10^,^70^ This model was shown to reproduce the correct impedance behavior across a wider range of frequencies. Numerically consistent ways to incorporate such downstream conditions within the 1D model has also been investigated.^104^ However, these methods require additional derivations and modifications to the implementation that are not always straightforward to apply. Therefore, a simpler solution of using a single tapered vessel as the outflow condition was proposed recently.^68^ This approach requires no additional implementation. The tapered vessel model has been used previously to approximate a whole arterial tree,^29^ and has the useful property of producing a series of reflected waves which are attenuated distally in a graded manner, as can be expected from a vascular tree.

Finally, in the standard model, the coupling of the vessel segments at a junction is normally achieved by two sets of equations. One imposes the conservation of mass and total pressure. These are supplemented by conditions on Riemann invariants of the 1D system^83^ or “compatibility” equations^32^ which prescribe appropriate conditions on the characteristics.

### Experimental Validation

The validity of using the 1D model for wave propagation studies in arteries has been investigated using a silicon experimental phantom of the systemic circulation, comprising 37 segments.^66^ Using measured rather than fitted material parameters, the 1D flow model yielded errors of less than 4% pressure and 19% flow for 70 measurements obtained over the network. Interestingly, the model was able to faithfully reproduce the frequency of the non-physiological oscillations introduced by the simple resistive boundary conditions. Further investigations revealed that the error in amplitude of the oscillations was not corrected by adding peripheral compliance, inertance or energy loss at branching junctions to the model. These authors suggested that the underdamping may be produced by neglecting the viscoelasticity of the tube walls, and later provided supporting evidences using a visco-elastic model of the network.^1^ This study was performed on a phantom, and its scale was significantly different to that of the coronary vessels. Nevertheless, its *in vitro* validation reasserts that the flow in an elastic tube network can be adequately modelled using the 1D model.

### Viscoelasticity of the Vessel Walls

It has long been established that vascular walls exhibit viscoelastic properties as evidenced by its typical hysteresis behaviour in load-unload cycles. The degree to which viscoelasticity plays a role in coronary flow remains to be fully ascertained; however, to date, modelling studies have offered several insights into this aspect. Pontrelli 77 used a coupled 1D-lumped outflow model to conduct a linearised wave propagation analysis. With periodical forcing, it showed that varying the elasticity coefficient modified the amplitude of the oscillation but had no effect on its frequency, while the viscosity of the wall played a predominant role in attenuating the high frequency wave components. This result lends support to the conclusion reached by the authors of the aforementioned *in vitro* experiment of Matthys^66^ and their more recent modelling work.^1^ An extended analysis was offered in Canic *et al.* 15 in which a more complete model description of viscoelastic vascular fluid–structure interaction was presented. Using the techniques of asymptotic homogenization, 3D Navier–Stokes and linear viscoelastic membrane equations were reduced to three systems of parabolic-hyperbolic equations, each of which required the computational complexity of a 1D system, but taken together, captured two-dimensional descriptions of axisymmetric flow features. In particular, the more complete description of the fluid viscous terms allowed the velocity profile in the viscoelastic vessel to be calculated as part of the solution. The model showed that the hysteresis behavior observed in the loading cycle of an artery is solely due to the viscosity of the wall, independent of the fluid viscosity. In addition, the in-depth treatment of the fluid viscous forces appears to indicate a greater contribution of fluid viscous effects to oscillation damping than was suggested by simpler models. Regrettably, this work lacked the description of junction coupling of vessels, limiting its usage so far to a single segment. Further developments in this direction would be useful in bridging the gap between full CFD and 1D models.

### Coronary System Modelling

Surprisingly, to date there has only been a limited number of attempts to study the transient pulsatile flow in a distributed 1D coronary network. An example of such an analysis is provided by Smith *et al.*,^87^,^88^ in which the regional myocardial stresses calculated with an anisotropic finite deformation model of the beating heart^69^ was applied on the vessels. The extravascular pressure applied to the vessels was calculated as the average radial stress on the local vessel. This approach unified the contributions from intramyocardial pump and varying elastance mechanisms (see *Earlier concepts* section), and contrasted the varying significance of each during diastole and systole. The phase difference between arterial and venous flow, and diameter variations of arterioles and venules corresponding to experimental observations in different myocardial layers were also reproduced.

However, although the above analysis captured the broad characteristics of coronary flow and its mechanical determinants, it was limited by two key aspects. The first is in its treatment of the microcirculation, which lumped the complex interaction between the flow-contraction crosstalk into a 0D resistance–capacitance model. The model did however capture their spatial distribution (and thus corresponding variation of myocardial stress) throughout the myocardial layers, as each lumped model was attached to the termini of the distal vessels. Secondly, due to imaging limitation (see next section) and thus the availability of anatomical data at the time of study, the vascular network used was stochastically generated rather than obtained from a sample. This may detract from the strength of the conclusions regarding the transmural variability of crosstalk mechanisms. Efforts to address both of these shortcomings are currently underway, with the poroelastic models reviewed below, and new advances in imaging of microcirculation (as previously reviewed^61^).

### Computational Solution Techniques

The numerical solution of the 1D flow equation system has been achieved using both finite difference^14^,^88^ and finite element techniques.^68^,^84^,^106^ Although the Lax-Wendroff finite difference method applied in previous studies^14^,^88^ achieved a good balance between accuracy and solution speed, the finite element method has been preferentially applied in more recent studies. Of note is the spectral/hp technique^83^ which is known to yield good dispersion and diffusion error properties well-suited for modelling wave propagation, and exponential ideal convergence rate.^48^ Techniques which can decouple the solution in each vessel have also been of particular interest, since they have the attractive property of avoiding large matrix inversion. In these schemes the information between vessels at a junction is passed through inter-element flux terms. Techniques of this type so far have employed discontinuous Galerkin^84^ and locally-conservative Galerkin formulations.^68^,^97^ With a nonlinear flow model, these methods may require an explicit time integration scheme to retain efficient segment decoupling. Additionally, if an inviscid flow is assumed, it is possible to decouple the mass and momentum equations into scalar equations by re-expressing them in terms of characteristic variables.^32^

## Poroelastic Modelling of Perfusion

In terms of both structure and function, it may be argued that the microvascular compartment is the most fundamental component of the coronary circulation. It is the site of major resistance (~70% with normal vasomotor tone^20^) and regulation of flow^52^ that is largely responsible for the coronary reserve which allows 4 to 5-fold increase in maximal flow rate during exercise. It is well-established that disease outcome correlates better with functional indicators (assessed by regional perfusion efficacy) than anatomical indices.^98^ The loss of vasodilatory capacity of the microcirculation (microvascular dysfunction) itself has been identified as a strong predictor of disease outcome and has emerged as a new therapeutic target.^13^,^64^ But while these factors strongly suggest that the coronary microcirculation deserves closer attention, modelling literature has been limited to date.

To model the spatiotemporal flow features in microcirculation with the approaches presented so far would be difficult; the acquisition of large and complex microvascular anatomy is onerous and would make the model computationally intractable. These difficulties motivate the application of a poroelastic modelling approach,^23^ which can capture the essential features of heterogeneous and anisotropic flow patterns without having to model the individual vessels. Furthermore, the approach naturally provides a framework to incorporate solid–fluid interactions i.e., direct and indirect crosstalk mechanisms at the microscale. The key idea underlying poroelasticity is in its treatment of mixtures which replaces the complex fluid–structure interaction with a superposition of both fluid and solid components. Using this framework a model description is obtained in terms of each homogenised component that now occupies a fraction of the volume in every point of the material. This can be achieved through a formal averaging process,^112^ which enables simpler geometrical descriptions and aggregates the microscopic interactions. Since its original development in soil mechanics,^3^,^4^ the poroelastic modelling approach has found its way into various applications in soft tissue mechanics. On the contrary, since the pioneering whole-heart poroelastic models of Huyghe *et al.* 42,47 only a limited number of studies have been proposed in the cardiac literature.

### Multicompartment Formulation

As mentioned above, in poroelasticity the microscopic variations of pressure and flow are averaged over a number of nearby pores. For a vascular system however this approach is inadequate since it would average together the pressures in the arterioles, capillaries and venules (typically in close proximity) meaning they would be impossible to distinguish. In order to address this issue an extended formulation has been proposed,^43^–^46^ using a “hierarchic parameter” which reflected the hierarchical position of the vessel segment with a normalised scale between zero and one. A prescribed interval of this parameter would then define a particular vascular compartment. Carrying this formalism through the derivation, the new set of fluid equations obtained additional coupling terms between the inter- and intra-compartmental flows. These new terms allowed the inter-compartmental pressure gradient to drive the intra-compartmental flows, and conversely intra-compartmental pressure gradient to drive the inter-compartmental flows.

Using the above formulation, Vankan *et al.* 103 conducted a numerical comparison between a finite element perfusion model and an explicit network flow solution obtained using the Poiseuille equation in a computer-generated vascular bed. Two alternative definitions for the hierarchic parameter were used, based on the vessel diameter or the pressure calculated from the solution of network flow. While qualitative agreements were found between the network and FEM model with either choice of the hierarchic parameter, the diameter-based compartmentalization resulted in a poor quantitative comparison. The requirement that vascular pressures must be known *a priori*, and the high sensitivity of the solution to this parameter present some practical difficulties. In a more recent multi-compartmental porous flow model^22^ a simpler formulation based on a double-porosity model^23^ was used. This model has the advantage of computational efficiency due to the collapsing of the hierarchical dimension to a few discrete lumped compartments, at the cost of providing a less general description. Despite this, a good agreement of spatial and compartmental pressure distribution was found between the network model and the continuum finite element model, applied in a pig left ventricular perfusion sub-region. Currently, the investigation to characterize the sensitivity and robustness of the approach is continuing.

### Calculation of Permeability

In porous perfusion models, the relationship between flow and pressure is usually expressed using the Darcy equation, which defines a linear proportionality between flow and pressure gradient. The proportionality constant (or function) is the permeability of the porous medium which is inversely proportional to the fluid viscosity. It is a tensorial quantity which is anisotropic for a typical vascular network, reflecting the material’s preferred directions of flow. A method to derive the multi-compartment permeability tensors from a set of circular, curved cylinders in arbitrary orientations was presented previously, along with their transformation under finite strain of the porous skeleton.^46^ It was assumed that fluid volume increase was distributed equally over the whole cylinder irrespective of its orientation. Additionally, as the hierarchical porous flow framework was used in its derivation, the permeability was a four-dimensional quantity that included off-diagonal terms to couple the intra- and inter-compartmental pressure gradients and flow. In comparison, an alternative approach was outlined,^22^ whereby multi-compartment permeabilities were calculated using a technique based on principal component analysis of straight cylinder networks. This method has useful properties such as guaranteed symmetry and positive definiteness, required for physical flow solutions, and also provides ease of extension, e.g., to diameter-dependent viscosity. Further extensions should elucidate the dependency on the averaging volume and shape, provide a validation of the multi-compartment flow and characterize the transformation of permeability under direct and indirect crosstalk with surrounding myocytes.

### Constitutive Behaviors

In the standard theory of hyperelasticity, the stress–strain relationship of the solid can be derived from a strain energy function. In a similar manner, thermodynamic analysis of poroelastic media leads to a free energy function from which state laws of solid (stress–strain) and fluid (mass-porosity) can be derived.^23^ Together, the equations describe the interaction between the solid and fluid phases. Chapelle *et al.*,^19^ developed a large strain poroelastic constitutive law to model cardiac perfusion, and presented a procedure to manage the singularity arising in the case where both solid and fluid phases are incompressible. Coupling this with an active stress generation model, an empty beating left ventricle was simulated that showed the effects of systolic flow impediment. In Cookson *et al.*,^22^ a large strain law was applied to simulate the stiffening behavior of the cardiac tissue with increased blood content. As the incompressibilities of solid and fluid phases were dealt with by using a Lagrange multiplier solution scheme, no specific procedure to address the singularity was required. Both of these constitutive laws are isotropic and empirical—currently a constitutive law which captures the fiber-sheet structure of the cardiac tissue^62^ and its inter-relationship with the embedded vasculature is not available. To develop a finite strain poroelastic law based on the cardiac microstructure, that is thermodynamically consistent, remains a major challenge for future applications of the porous modelling approach.

## Three-Dimensional and Multiscale Models of Coronary Flow

Although mechanical crosstalk and perfusion have comprised the central theme of this review so far, there are important reasons to examine the detailed local flow features in larger vessels of the coronary network. After all, atherosclerotic plaques are found most commonly in the proximal vessels, and localised to major bifurcations and inner walls of curved segments. The correlation between low and oscillatory wall shear stress (WSS) and atherosclerosis^16^ has been the subject of intense research. As the shear stress distribution is highly sensitive to individual vessel geometry, detailed CFD approaches have been employed to characterize WSS distribution and reveal potential sites and determinants of vulnerability. The state of the art in large multiscale image-based modelling of arterial networks has been detailed in recent reviews.^36^,^96^ Although most of these applications were in non-coronary vessels, the tools and techniques applied therein are directly relevant to the coronary circulation. On the other hand, the dynamic motion of the myocardium and fluid–structure interaction with surrounding myocytes introduce increased computational challenges in coronary CFD models.

### Patient Specific Coronary Modelling

Unfortunately the scope of this review does not permit an in-depth description of mechanistic CFD studies in the coronary vessels, however, an increasing number of work in recent years have employed CFD to examine patient-specific flow characteristics in coronary arteries. Biplane-cineangiography has been commonly used to reconstruct 3D coronary geometries, and can be acquired during a routine catheterization procedure with no additional equipment or training. Coronary CFD has been combined with a surface area based categorization of WSS (akin to the ideas expressed previously^33^,^91^) and statistical analysis in a small set of patients to discriminate between healthy, obstructive or aneurysmatic coronary artery disease with high accuracy.^107^,^108^ Also, a completely non-invasive subject-specific imaging-based CFD study was recently performed.^99^ The vessel geometry, dynamic motion and proximal velocity waveforms were all acquired using custom designed MR protocols. CFD based on an arbitrary Lagrangian–Eulerian formulation yielded consistent results with previous literature which demonstrated the potential of MRI in patient specific coronary CFD modelling. Unfortunately however, the study was severely limited by its long image acquisition time (7 scanning sessions in total), inordinate even for routine research work. Although MRI-based coronary CFD remains a feasibility-only study for now, new advances in MR coronary imaging could be expected to broaden its scope in future.^21^

### Integrated Multiscale Modelling

Owing to the enormous computational demands of the 3D CFD models, the brute force simulation of a complete coronary circulation is currently intractable, and will remain so in foreseeable future. A recent study which compared 3D and 1D simulations of 50 intracranial arteries^37^ reported a 24 h running time on 256 cores per cardiac cycle for the full model. In comparison the 1D counterpart required only 20 min per eight cardiac cycles on a single-core desktop, which is a ratio of approximately 150,000. There are also questions as to whether a model that includes all individual vessels of the microcirculation can be correctly parameterised, and whether experimental and modelling assumptions made at various stages will begin to dominate the outcome. A solution to the tractability problem was first proposed soon after image-based blood flow modelling began. It involved the use of a heterogeneous set of 3D, 1D and lumped parameter models that were coupled as a means to tackle the hierarchical vascular flow.^32^ This idea has been applied in many subsequent studies,^8^,^57^,^67^,^100^,^101^ including those which included 3D coronary vessel flow as part of a closed-circuit whole body circulation.^50^ The mathematics of coupling the multidimensional models (e.g., parabolic-hyperbolic PDEs in the case of 3D-1D coupling or PDE-ODE for 3D-0D coupling) is non-trivial, as incompatible models at the interface may introduce discontinuities in the solution variables. Additional assumptions must also be made to address the degeneracy between the full 3D field and averaged quantities of the reduced-order model at the interface. The well-posedness of the 3D-0D coupling has been previously analyzed,^80^ while numerous approaches for the 3D-1D problem have been proposed, beginning with the iterative coupling strategy of Formaggia *et al.* 30,31 This work was followed by an implicit coupling strategy, in which the downstream impedances were taken into account through a Dirichlet-to-Neumann mapping^105^ that provided more stable convergence behavior at the cost of modifications to the governing equations. A further generalization was made in an extended variational formulation,^6^ which was applied in subsequent numerical simulation studies.^7^,^8^

## Wave Intensity Analysis and Real-Time Clinical Application

While multiscale models of coronary flow have yet to make a significant impact in the clinical environments, recent achievements exploiting the wave intensity analysis (WIA) techniques serve as an exemplar and an elegant demonstration of application of modelling in real-time clinical diagnosis. Although WIA shares common theoretical foundations with the1D fluid models, its philosophy can be contrasted with the multiscale approaches presented so far, as it is applied to analyze waveforms acquired in a point-wise manner in practice. The applications of WIA in coronary circulation and its role in clinical developments are briefly described below.

### Wave Intensity Analysis

Wave intensity analysis is a time-domain analysis technique which is applied to arterial pressure and velocity waveforms. Pioneered close to 25 years ago by Parker *et al.*,^73^,^74^ the theoretical background of WIA and an overview of its subsequent applications can also be found in recent reviews.^41^,^72^ The application of WIA in the coronary circulation has a relatively recent history, but has yielded substantial new understandings. By building upon theoretical foundations of method of characteristics, WIA has provided a series of insights relating the pressure and velocity waveforms to the propagation speed and intensity, and allowed a separation of forward and backward traveling waves.^72^ By separating the forward and backward wave components, Sun *et al*.^93^ demonstrated that epicardial flow can be interpreted in terms of the interplay between upstream (aortic, forward-traveling) and downstream (microvascular, backward-traveling) sources of wave origination. Various cardiac events such as opening/closing of the aortic valve or the onset of systole/diastole would generate a propagating pulse wave which increases or decreases the arterial pressure and velocity. This concept has been extended to an animal study to draw a quantitative relationship between backward compression wave intensity and systolic impediment.^94^ A later study offered a more comprehensive description, characterising the human coronary circulation with six predominating waves.^24^ It also found that 94% of the forward acceleration was due to two waves generated by LV ejection (forward) and microvascular de-compression (traveling backwards toward aortic sinus). Interestingly, the latter ‘backward-traveling suction wave’ was found to be responsible for a much larger portion of the anterograde flow acceleration.

### Fractional Flow Reserve and Instantaneous Wave-Free Ratio

Fractional flow reserve (FFR) is a clinical index acquired during a catherization procedure that estimates the functional severity of a coronary stenosis. It is a strong indicator of ischemia, and was developed to overcome the poor diagnostic values of purely anatomical measures in assessing the functional consequences of stenoses, particularly those with intermediate severity.^5^,^26^ It is defined as the ratio of maximal blood flow in a diseased artery to maximal flow if that same artery was to be normal.^76^ One of the assumptions critical to FFR is the invariant resistance of the microvascular bed supplied by the artery, between healthy and diseased states. To ensure this condition, vasodilating agents such as adenosine are administered during catheterization. Although its theoretical foundation is dependent on a number of idealised assumptions, a substantial body of clinical evidence has validated its value in guiding whether a coronary intervention should be carried out for improved patient outcomes.^98^ Despite this, the clinical adoption rate had been poor, owing mainly to the need for adenosine-induced hyperemia, which increases the procedure time and cost.

The instantaneous wave-free ratio (iFR) has recently been proposed as an alternative stenosis severity index which requires no pharmacological administration. iFR is defined as the instantaneous ratio of translesional pressures, acquired during a specific period of diastole, named the wave-free period. A recently completed pilot clinical study^82^ provided evidence that during the wave-free period, microvascular resistance is similar to that achieved using pharmacological means. iFR acquired during this period was found to be strongly correlated with FFR, exhibiting high positive and negative predictive values and reproducibility. WIA has also contributed to the usage of iFR in practice in ancillary ways, though the development of a new wave velocity estimation method. In applying WIA in a vessel, an estimate of the wave velocity is required. In a vessel as short as the coronary segment, the traditional two-point foot-to-foot measurement method can be difficult to apply in a reliable manner. The development of a one-point wave speed measurement technique,^25^ which uses the recently developed dual pressure–velocity sensor guidewire,^86^ was instrumental in addressing this issue.

## Conclusions and Future Outlook

Building upon the concepts of coronary-cardiac interaction established by earlier studies, the one-dimensional flow modelling has reinforced the importance of wave propagation phenomena and contributed a number of additional insights into the factors governing coronary flow. Further efforts are continuing in detailed CFD and multiscale models, which gained recent momentum, to produce models that are both subject-specific and predictive. However, it must be stated again that even though the techniques reviewed here set the stage for multiscale flow modelling, coronary flow cannot be studied in isolation to the rest of cardiac function. Specifically, the studies of mechanical crosstalk will require regional and quantitative descriptions of myocardial stresses, which currently is attainable only through a model simulation. Thus an integrated cardiac model including myocardial contraction, systemic circulation, ventricular fluid–structure interaction and electromechanical coupling^59^ are of paramount importance to the simulation of coronary flow. The continuum description adopted by the poroelastic approach is well-suited to the existing framework of integrated cardiac modelling. Future efforts should also address the flow in coronary microcirculation as much remains unknown regarding the precise role and etiology of microcirculation in diseases. Modelling flow in the microcirculation presents a new series of challenges including autoregulatory responses,^65^ rheological complexities^78^ and structural adaptation and angiogenesis^75^ in addition to anatomy and crosstalk. Relevant modelling studies in the microcirculation have been outlined in a previous review.^61^

Regarding clinical translation, the wave intensity analysis technique has undoubtedly made profound contributions to diagnosis of coronary heart disease within a relatively short time. However it must be reminded that such progress was enabled by over two decades of prior research, and that these developments do not represent the end point; WIA is a globally aggregated approach and thus cannot be used to study regional or transmurally varying coronary phenomena. As commented previously,^85^ the ability of WIA in assessing myocardial perfusion has not yet been established. It would appear that the application of coronary models to clinically-oriented studies remains limited in comparison to other vascular models e.g., carotid,^58^,^95^ cerebral aneurysms^17^,^79^ and abdominal aortic aneurysms.^63^ While the causes for this are multifactorial, the present inability of the imaging technology to access the small intramyocardial vessels and the complex physiology of the heart are undoubtedly contributing factors. On both accounts we see forthcoming opportunities—the new advances in MRI technology is poised to attain the elusive *in vivo* non-invasive imaging of the regional perfusion assessments,^35^,^39^ while the need to understand the physiological and pathophysiological mechanisms will propel new joint efforts between modellers and experimentalists in basic science. Furthermore, we remark that in order for multiscale coronary modelling to gain clinical momentum the lessons of iFR—that a degree of crudeness in modelling can be tolerated and still achieve useful real life applications—need to be heeded, and encourage further translational applications of the modelling technology in this vein.
